# Reinforcement Learning and Its Clinical Applications Within Healthcare: A Systematic Review of Precision Medicine and Dynamic Treatment Regimes

**DOI:** 10.3390/healthcare13141752

**Published:** 2025-07-19

**Authors:** Timothy C. Frommeyer, Michael M. Gilbert, Reid M. Fursmidt, Youngjun Park, John Paul Khouzam, Garrett V. Brittain, Daniel P. Frommeyer, Ean S. Bett, Trevor J. Bihl

**Affiliations:** 1 Department of Internal Medicine, The Ohio State University College of Medicine, Columbus, OH 43210, USA; 2 Department of Radiology, The Ohio State University College of Medicine, Columbus, OH 43210, USA; 3 Northwell Health System, Department of Radiology, Zucker School of Medicine at Hofstra/Northwell Manhasset, New York, NY 11549, USA; 4 Boonshoft School of Medicine, Wright State University, Dayton, OH 45324, USA; 5 Department of Pharmacology & Toxicology, Boonshoft School of Medicine, Wright State University, Dayton, OH 45324, USA; 6 Department of Biology, University of Dayton, Dayton, OH 45469, USA; 7 Department of Family Medicine, Fairfield Medical Center, Fairfield, OH 43130, USA

**Keywords:** reinforcement learning, machine learning, artificial intelligence, precision medicine, dynamic treatment regimen, health informatics

## Abstract

**Background/Objectives**: Reinforcement learning (RL), a subset of machine learning, has emerged as a promising tool for supporting precision medicine and dynamic treatment regimes by enabling adaptive, data-driven clinical decision making. Despite its potential, challenges such as interpretability, reward definition, data limitations, and clinician adoption remain. This review aims to evaluate the recent advancements in RL in precision medicine and dynamic treatment regimes, highlight clinical fields of application, and propose practical frameworks for future integration into medical practice. **Methods**: A systematic review was conducted following PRISMA guidelines across PubMed, MEDLINE, and Web of Science databases, focusing on studies from January 2014 to December 2024. Articles were included based on their relevance to RL applications in precision medicine and dynamic treatment regime within healthcare. Data extraction captured study characteristics, algorithms used, specialty area, and outcomes. **Results**: Forty-six studies met the inclusion criteria. RL applications were concentrated in endocrinology, critical care, oncology, and behavioral health, with a focus on dynamic and personalized treatment planning. Hybrid and value-based RL methods were the most utilized. Since 2020, there has been a sharp increase in RL research in healthcare, driven by advances in computational power, digital health technologies, and increased use of wearable devices. **Conclusions**: RL offers a powerful opportunity to augment clinical decision making by enabling dynamic and individualized patient care. Addressing key barriers related to transparency, data availability, and alignment with clinical workflows will be critical to translating RL into everyday medical practice.

## 1. Introduction to Reinforcement Learning and Its Applications

### 1.1. Introduction

Artificial intelligence (AI) makes use of computer-based technology to augment and increase human understanding and capacity. Machine learning (ML) is a subdiscipline of AI, describing how computers learn to associate and provide predictive power through datasets. Collectively, the application of AI/ML in medicine is being seen in a wide range of medical subspecialities including dermatology, oncology, epidemiology, gastroenterology, and cardiology [1,2,3,4,5]. By training on data, an AI tool responds to new data based on similar frameworks and is able to undertake complex tasks [6,7], allowing for new opportunities to improve patient care, optimize health systems, and transform medical subspecialties. Of the many challenges in this field, the most important may be creating translatable and actionable processes and converting computer science-based technologies into applications clinicians can understand and make clinical decisions from. However, ML has already shown increasing efficacy in the medical field in terms of diagnosis and outcomes prediction. Furthermore, its employment within healthcare is promising given its ability to make accurate predictions using various sources of data [7].

**Figure 1:**
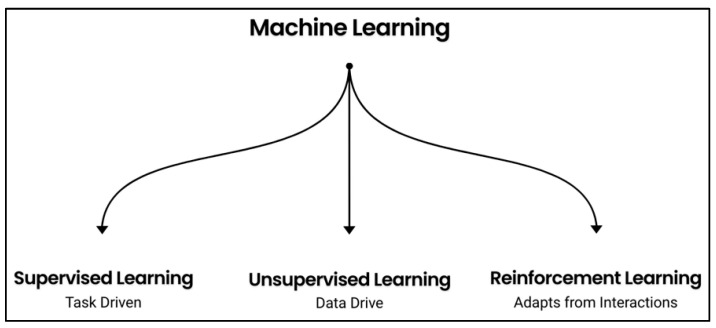
The three approaches to machine learning: supervised learning, unsupervised learning, and reinforcement learning. Their broad applications in medicine are noted.

There are primarily three approaches to machine learning: supervised learning, unsupervised learning, and reinforcement learning (RL), all of which have applications in medicine (Figure 1) [8]. Supervised learning makes use of a dataset that includes a labeled input and output class or “target” at the start of training, in effect creating a predictive model (through classifications or regressions) to help with future projections on fresh data. This process has been used in practices ranging from disease prediction to diagnosis, prognosis, and staging in oncology [9,10]. Unsupervised learning differs from supervised learning because it uses an unlabeled dataset with no predefined or desired output. It often utilizes clustering to find input regularities and to reduce dimensionality. This approach has been employed in a wide range of medical applications including radiomics, pathology, and the musculoskeletal system [11,12,13].

Reinforcement learning is policy-based and focuses on solving problems where there is an interaction between an agent (which produces an action) and the environment (which provides a specific reward or penalty) [8], enabling the model to identify the most effective way to achieve an intended result. The applications of RL within medicine and healthcare are broad and diverse. It is starting to be utilized more in clinical fields including internal medicine, surgical subspecialties, and healthcare administration [14,15,16,17]. Prior reviews have grouped the implementations of RL into categories, with minor variations depending on the source [16,18,19,20,21]. They are as follows: precision medicine (PM), dynamic treatment regime (DTRs), personalized rehabilitation, medical imaging, diagnostic systems, control systems, dialog systems, clinical support systems, health management systems, drug discovery and development, robotics-assisted surgery, and wearable devices and remote patient monitoring (Table 1). Compared to traditional rule-based systems and many supervised ML methods, RL offers a distinct advantage in its ability to optimize sequential decision-making through dynamic feedback. This makes RL well-suited for clinical care and other applications, including treatment planning, dosage titration, and personalized regimens, where actions must adapt over time to evolving patient responses.

In this paper, we focus on the applications of RL in medicine in regard to PM and DTRs. These two categories were chosen because of their focus on personalized, adaptive, and data-driven decision making, the hallmark of RL applications. The authors believe this to be the catalyst for the next evolution of data-driven medicine. We also provide a special focus on the ways physicians and other clinicians may use RL in daily practice, including the clinical know-hows and practical implications. Further, we will discuss current challenges, and future directions that are applicable for medical clinicians being introduced to this topic. Finally, we will provide examples that illuminate how RL has the potential to be directly integrated into the clinical practice with minimal friction, thereby allowing physicians of any background or with a limited knowledge base to take part in its implementation. This paper differs from prior comprehensive reviews by our focus on PM and DTR, as well as providing practical knowledge for clinicians [16,19,20,21,22].

### 1.2. Background of RL

AI includes a wide variety of computational means to create artificial inference. Definitions of AI vary along two main dimensions, the first is concerned with thinking versus acting, and the second dimension is concerned with whether the standard is to emulate humans or to achieve rationality [23]. This yields four categories of AI: (1) systems that think like humans; (2) systems that act like humans; (3) systems that think rationally; and (4) systems that behave rationally. The fourth category is arguably the most valuable category to consider for AI development for several reasons. The first reason is that inference is one of many mechanisms that can be used for achieving rational behavior. Secondly, rationality is more compliant to scientific systems than systems that mimic humans. Thirdly, it is intrinsically difficult to understand the thinking process, but behaviors are measurable. All together, these characteristics are important to an end user. For the focus herein, a physician end user always needs to consider and process real-time information and decide the best rational decision for their patients.

**Figure 2:**
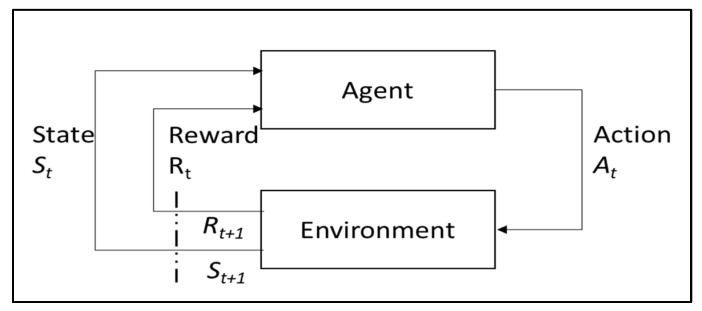
General conceptualization of reinforcement learning.

RL has served as a paradigm for solving very complex decision-making problems because of several high-profile successes in various problems [24]. Notable non-medical examples include general Atari 2600 game-playing, control of robots, and the famous defeat of a top human expert at the game of GO [25,26,27]. RL problems are specified in terms of a state space, an action space, and a reward function [28]. As the agent takes actions, the environment state changes and a reward is received. The agent aims to maximize the cumulative reward by learning an appropriate policy which works in the given environment. An example of the general RL process is illustrated in Figure 2. Depending on the algorithmic approach, the RL agent will have at least one of the following components: a model, a policy, or a value function [28]. A model can be mathematical models of the agent’s dynamics and rewards. A policy maps a state to an action, or a strategy for deciding what action to take in a given scenario. Lastly, a value function accounts for the future rewards the agent will accrue by being in certain states and taking the specified actions according to a particular policy [28].

These three components (value functions, policies, and models) underlie three general approaches to solving RL problems: methods based on value functions, methods based on policy search, and model-based methods [23,29]. Model-based methods allow the agent to plan based on its model of the situation, but often a complete ground truth model (a model that represents an accurate and definitive standard against which other models or predictions can be validated) is usually not available. This prompts the need for model-free approaches based on the policy or value function. Policy-search methods allow for an agent to directly learn a policy, which dictates what actions to take from any given state. Value-based methods only indirectly optimize for agent performance but can be substantially more efficient. Value-based methods and policy search methods are not exclusive to one another. A common technique is to combine both value-based methods with policy search methods to form value–policy hybrids, for example actor–critic methods [23,24,29]. More recently, deep RL has been developing in medicine. Deep learning involves the utilization of a set of inputs and transforms it into output using an artificial neural network. Deep RL combines a deep learning algorithm with RL, which allows agents to make decisions from unstructured input data similar to the way a brain would make a decision in an RL framework [30]. In their daily practice, doctors frequently use frameworks like those of RL in which parts of the actions they perform are learned through trial-and-error interactions with their patients. Even seasoned doctors occasionally encounter novel pathologies, requiring them to experiment in their environment.

### 1.3. Research Directions

While the potential of RL has been demonstrated in robotics and successful applications to games such as Go and Atari, its use within healthcare is more complicated due to the nature of clinical practice. Regardless, RL has received significant attention within the medical community due to its ability to support precision medicine, learn about clinical treatments, discover new medical knowledge, and ascertain patterns in clinical data [19].

Currently, RL research in medicine is focused on overcoming the heterogeneity within patient populations. No two patients are exactly alike, given their comorbidities, family histories, real-time diagnostic testing, and many other factors. Individualized treatment plans are increasingly utilized to help produce optimal healthcare, particularly for diseases like Parkinson’s, cervical cancer, and type 2 diabetes [17,31,32]. These pathologies, among many others, require continuous complex decision-making processes over a long course of time. RL is uniquely equipped to handle these tasks.

Often, these RL applications are aimed at addressing complex decisions in the form of modifying previously static dosing regimens. Dosing requires constant analysis into an array of factors not limited to weight changes, kidney function, liver function, burden of treatment on the patient, and overall health and energy. This is where RL research and wearable devices come together for the continuous examination of patient symptoms. For example, one study utilized wearable sensors on patients with Parkinson’s disease to examine bradykinesia and dyskinesia. This helped in evaluating the best time to administer Parkinson’s medication and in determining the dose at which symptoms are minimized [31].

The current landscape has largely focused on fields such as oncology to optimize treatment plans, generating overall better-quality regimens with fewer symptoms and extended survival [33,34,35]. Unlike the long-term pathologies outlined above, acute illnesses like sepsis require immediate action. Sepsis management is a great candidate for RL research because it possesses multiple simultaneous inputs requiring constant monitoring of a patient’s health to ideally manage their critical needs. As a result, several recent studies examined the exact methodology of intervention delivery based on the patient’s unique profile and state [36,37,38,39,40]. Ultimately, the current goal of RL research for medical applications is to minimize the decision-making burden on clinicians without compromising patient outcomes and to highlight time-critical opportunities in the treatment process. This is similar in concept to many traditional RL applications which aim to coordinate operations to focus human intervention to the most impactful level [29].

RL holds significant promise in enhancing PM and DTR within medicine and healthcare. Their focus on personalized, adaptive, and data-driven decision making enables them to evolve care while accounting for a patient’s needs and responses over time.

#### 1.3.1. Introduction to Precision Medicine

**Figure 3:**
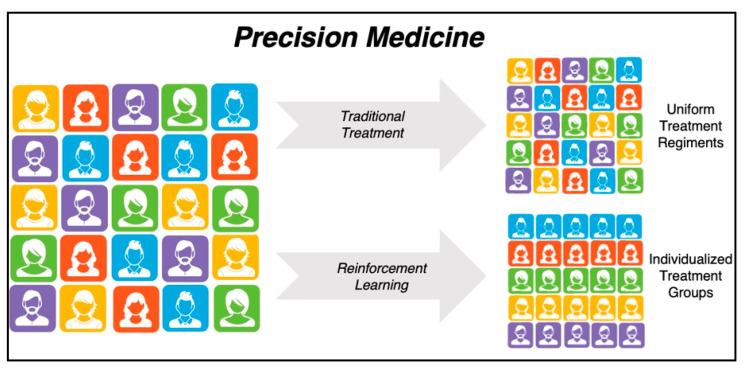
Reinforcement learning and its approach to precision medicine.

PM is sometimes referred to as personalized medicine and has been defined as “the tailoring of medical treatment to the individual characteristics of each patient” [41,42]. Within the current healthcare system, medical decisions are often influenced or based on national guidelines (i.e., USPSTF, ACC/AHA, etc.). The recommendations are commonly based on evidence from high-quality randomized control trials and large population cohort studies. However, these traditional static approaches often rely on predefined protocols that are applied within heterogeneous patients. PM is enhanced from RL because it offers a way to continuously optimize treatment plans in real time by adapting to patient feedback and outcomes (Figure 3). This is made possible through RL’s ability to analyze patient data (such as vital signs, laboratory values, medical history, imaging, genetic information) and learn the optimal intervention strategies based on how a patient responds to the treatment [43]. This allows the model to personalize treatments rather than relying on generalized clinical guidelines, providing further benefits in diverse patient populations. Applications of RL within PM are broad, including surgery, internal medicine, and public health [15,16,44,45]. Furthermore, unlike the one-size-fits-all protocols, RL models continuously refine treatment strategies based on evolving patient conditions—a closely related phenomenon of DTR.

#### 1.3.2. Introduction to Dynamic Treatment Regime

DTR is closely related to PM. As defined by Chakraborty et al., DTR “consist of a sequence of decision rules, one per stage of intervention, that dictate how to individualize treatments to patients based on evolving treatment and covariate history” [46]. These “regimens” fit within a larger paradigm of PM. More specifically, PM dictates what treatment to provide based on a patient’s state. DTR expands the concept of personalized medicine to encompass evolving treatment settings, where care is continually adjusted to align with a patient’s dynamic and time-varying state. This application of RL has strengths in adapting to disease progression or handling complex and multistep treatments. For example, in diseases like cancer or chronic conditions, RL can optimize the treatment course by adjusting pharmacotherapy based on how the disease responds, while also working to minimize side effects [47,48]. In addition, many healthcare conditions, such as sepsis or insulin management, require multistep treatments over a period of time [49,50]. RL models are well equipped to manage this complexity versus conventional modes.

### 1.4. Current RL Challenges

The biggest challenge when it comes to RL in medicine is parallel to general issues in RL research: extrapolating to new problems and appropriately maximizing reward. For many, the “reward” in medicine means enhanced survival, yet that is not the only relevant endpoint one must consider when designing a treatment plan. Other things one must consider include tolerability, ease of compliance, cost, and quality of life. RL applications also need to ensure rewards gained are legitimate and not from hallucinatory “reward hacking” results, whereby ill-posed reward functions enable rewards to be accumulated through physically nonsensical means [51].

Thus, a significant challenge for RL moving forward is the definition of reward for clinical applications, which ultimately may result in outputs that do not necessarily coincide with those of the patients or those of the clinicians. Even more nuance is brought into this idea of reward when examining short-term versus long-term outcomes [52]. For example, “reward” in the short-term can directly contradict long-term reward when examining states like disease remission. In other words, a treatment that does not yield benefit immediately may in fact better suit patients when combined with another agent rather than a single treatment with immediate success [52]. Sequence in treatments matters and these trade-offs of short-term gain versus long-term success are difficult for RL to overcome, as they are for clinicians.

Another challenge for all RL is lack of transparency. Such issues run counter to the general desire for an understanding of the decision-making process in medicine. However, as the potential benefits and uses of RL become more widespread within medicine, the ‘nuts and bolts’ of what makes up any individual RL algorithm will inevitably become more unclear to clinicians, which is another large hurdle facing RL implementation. If a clinician is unable to say for certain what inputs were used, what reward was being examined at the highest cost, or what outputs versus traditional treatments were measured, then we must ask: are clinicians really able to implement such a tool?

A key hurdle that also affects the use of RL in medicine concerns data acquisition and computation. For many of the rare pathologies that exist, clinicians are limited by the available patient cases and data. For instance, twenty percent of all cancers diagnosed in the United States are defined as rare cancers [53]. The question then becomes the following: are there enough patients to enable an RL algorithm to be made which can enhance clinical treatment? This may lead many to face the infamous curse of dimensionality, which refers to the exponential increase in the number of possible actions as the number of features (degrees of freedom) increases [54]. RL takes a large amount of data to train on and learn from, raising the computational cost significantly. The worry is that clinicians do not have the resources to execute such a workflow [54]. Lastly, when considering RL, the mere reproducibility of the data is an important factor; there are limited benchmarks available for ensuring reproducibility from an existing RL algorithm. This is due to several factors, including the intrinsic variance of any individual algorithm, the stochasticity of the environment, and the dependence on several hyperparameters [55]. This is not to say that it is impossible to reproduce any given RL algorithm; however, there needs to be standardized benchmarks by which others can reproduce the RL algorithm.

## 2. Systematic Review

### 2.1. Methodology

**Figure 4:**
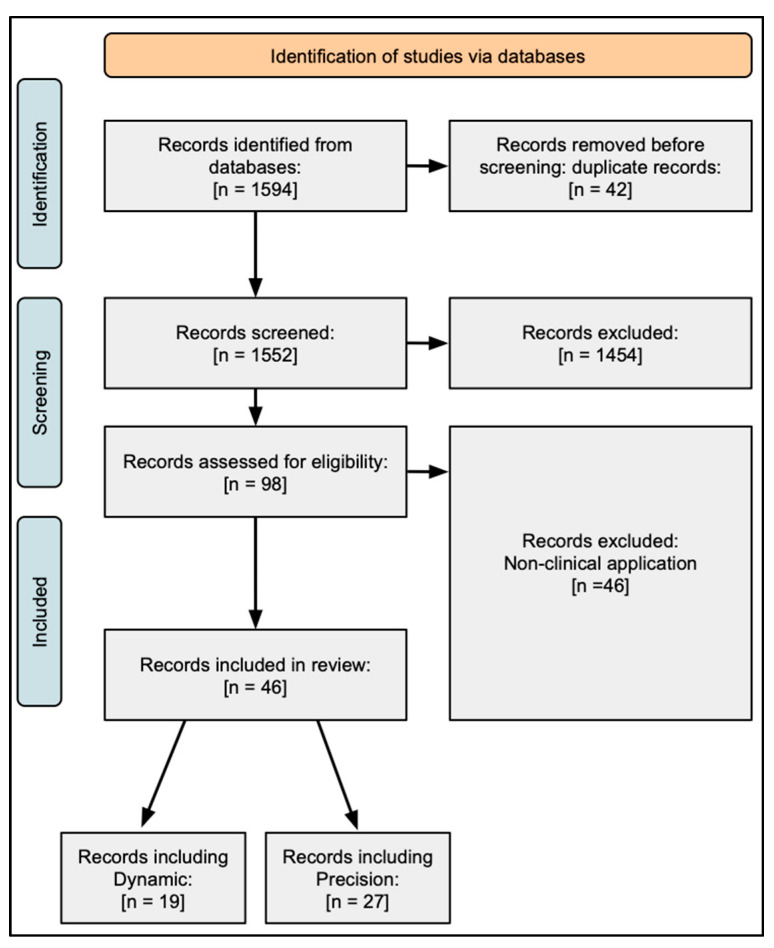
PRISMA flow diagram of literature review and article selection.

This systematic review was conducted following the PRISMA (Preferred Reporting Items for Systematic Reviews and Meta-Analyses) guidelines. A comprehensive literature review was conducted across several databases, including PubMed, MEDLINE, and Web of Science. Other bibliographic databases (such as IEEE Xplore and arXiv) were not included, as this scope was chosen to focus on the studies that are most relevant to clinical practice. All publications regarding the applications of RL within dynamic treatment regime and precision medicine published in the last 10 years (January 2014–December 2024) were identified. The final search strategy for publications within each database was as follows: (Reinforcement Learning AND (Dynamic Treatment OR Precision Medicine)). A total of 1594 articles were identified through initial screening, with retrieved references imported into EndNote for deduplication and screening. Inclusion criteria included papers only related to the application of RL versus other ML algorithms, uses of RL within dynamic treatment regimen or precision medicine, articles published within the past 10 years, articles published in English with full-text available, impact and relevance of the paper, and RL applications within healthcare. Articles were excluded based on not meeting the inclusion criteria, priority focus on other ML algorithms, focus on alternative RL categories, insufficient data size for algorithm training and validation, and other publication types, including commentaries, editorials, case reports, systematic reviews, and meta-analyses. The team utilized three screeners to limit bias and resolve any screening discrepancies in the literature review and selection. After the assessment of the available papers, 46 articles were selected for analysis and inclusion within the review (Figure 4). A summary of excluded full-text studies and reasons for exclusion is provided in the Supplementary Materials, Table S1.

**Figure 5:**
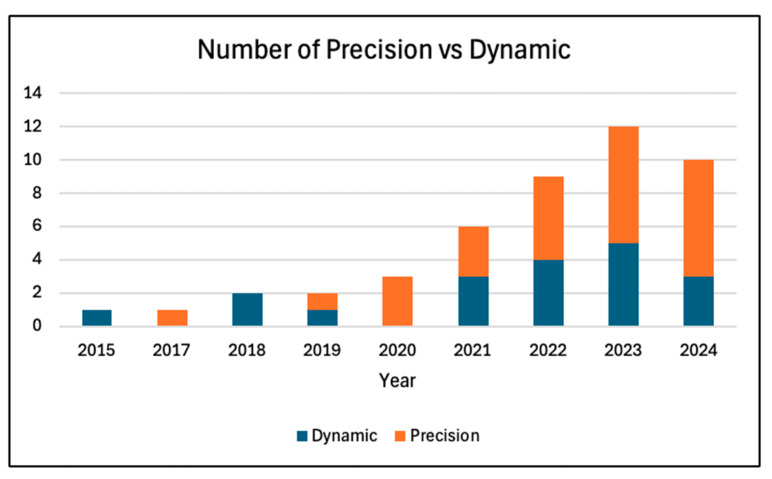
Number of papers arranged by reinforcement learning category.

**Figure 6:**
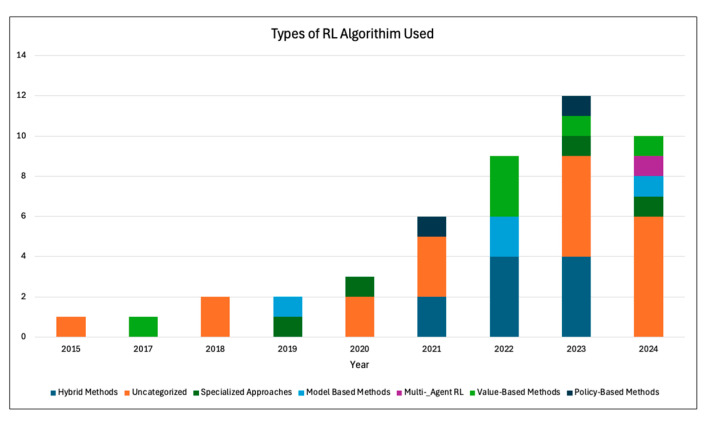
Number of papers arranged by reinforcement learning algorithm.

**Figure 7:**
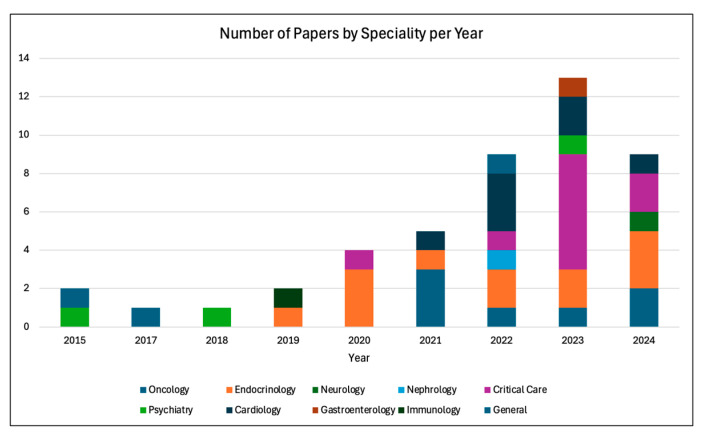
Number of papers arranged by specialty.

Data extraction was conducted independently by two reviewers using a predefined extraction form. The extracted information included article characteristics, study methodology and data analysis, medical specialty, ML algorithms used, clinical applications, outcome measures, and results. For quality assessment, the analysis was completed by two reviewers. The overall quality of the evidence was evaluated further using the GRADE (Grading of Recommendations, Assessment, Development, and Evaluation) framework, represented by Table S2 in the Supplementary Materials. An analysis of the papers was conducted, including a distribution of the surveyed papers in regard to their category, RL algorithm, year of publication, and impact. The included papers are further discussed and highlighted in the review. No quantitative effect measures were calculated. Due to heterogeneity in outcomes and study designs, a narrative synthesis was conducted. No data conversions or imputations were required. Study results were summarized narratively and supported by descriptive figures (Figure 5, Figure 6 and Figure 7). No statistical meta-analyses, heterogeneity analyses, or sensitivity analyses were performed due to diversity in study designs, outcome measures, and study aims. Reporting bias was not formally assessed as no quantitative synthesis was performed. As such, results regarding reporting bias were not applicable. This systematic review was retrospectively registered on the Open Science Framework (OSF) and is publicly available at https://doi.org/10.17605/OSF.IO/8fRJP (accessed on 12 July 2025).

### 2.2. Dynamic Treatment Regime

DTR provides a robust and innovative framework for optimizing patient care by adapting therapeutic interventions in real time to an individual’s evolving health state. This approach facilitates the delivery of personalized, multistage treatments designed to address complex, chronic, or acute conditions with improved outcomes. The dynamic and adaptive nature of DTR is particularly valuable in medical scenarios where patients experience fluctuating or progressively deteriorating conditions. By leveraging DTR, clinicians can fine-tune treatment strategies to align with a patient’s unique and changing needs, offering a new level of precision in healthcare delivery. This framework has demonstrated its utility across a variety of fields, from chronic neurodegenerative diseases to acute critical care settings.

In the field of neurology, DTR has proven particularly impactful for certain diseases such as Parkinson’s disease (PD), a progressive brain disorder characterized by debilitating motor symptoms. Patients with PD often experience transitions between the so-called ‘ON’ and ‘OFF’ states, where medications, such as dopaminergic agents, are effective only during the ‘ON’ state. A study conducted by Shuqair et al. highlighted the potential of reinforcement learning in this domain [56]. The researchers developed an integrated deep Long Short-Term Memory (LSTM) neural network coupled with multiple one-class unsupervised classifiers to create an RL-based adaptive classifier. This system was tested using two datasets of PD patients, enabling the model to accurately predict periods when medication would be most effective. Such advancements in RL-based classifiers have the potential to significantly improve the quality of life for PD patients by personalizing treatment timing to maximize therapeutic efficacy while minimizing adverse effects.

While neurodegenerative diseases benefit from RL-guided interventions that optimize medication timing, other conditions and specialties, such as psychiatry, can benefit from RL. Substance use disorders (SUDs) require adaptive treatment strategies that address long-term behavioral patterns. SUDs represent chronic and multifactorial conditions where maintenance of remission and prevention of relapse are critical components of treatment. Tao et al. introduced a tree-based reinforcement learning (T-RL) method that employs a recursive, tree-based approach to dynamically adjust treatment decisions over time [57]. This approach allows the model to consider the evolving behavioral patterns of individuals, resulting in tailored and effective treatment plans. For instance, T-RL has demonstrated the ability to adapt to changes in a patient’s behavior, providing more accurate interventions as the patient progresses through recovery. Another study by Zhao et al. explored the use of two distinct DTRs, Backward Outcome Weighted Learning (BOWL) and Simultaneous Outcome Weighted Learning (SOWL) in the context of smoking cessation [58]. Both approaches utilized multistage personalized treatment frameworks, enabling tailored interventions based on patient responses over time. When compared to fixed, non-adaptive strategies, these DTR models showed significantly better outcomes, highlighting the advantages of using RL to address the complex behavioral dynamics associated with SUDs. The success of these models underscores the potential of DTR to transform the management of chronic behavioral health conditions.

In addition to long-term interventions, DTR has also shown promise in fields that manage acute, life-threatening conditions such as sepsis. Sepsis is a leading cause of mortality in intensive care units (ICUs) and is characterized by a dysregulated immune response to infection, leading to life-threatening organ dysfunction. Effective management of sepsis requires rapid, evidence-based decisions to address the patient’s deteriorating condition. Bologheanu et al. utilized the Markov Decision Process (MDP), a commonly used RL framework, to optimize corticosteroid dosing in septic patients [59]. Analyzing data from 23,106 ICU admissions, their study revealed that RL-driven optimization of corticosteroid timing and dosage significantly reduced ICU mortality. Similarly, Zhang et al. developed a goal-oriented reinforcement learning (GORL) model tailored to the management of sepsis [60]. This approach addressed two critical challenges in RL applications for ICU patients: the delayed rewards inherent in sepsis treatment and the complexity of patient states. This model demonstrated a 10.23% reduction in patient mortality, further emphasizing the life-saving potential of RL in acute care settings.

Beyond the realm of critical care, DTR has been applied to the field of cardiology. One of the most common and life-threatening conditions to manage is coronary heart disease, a condition that often requires dynamic medication adjustments in response to acute hemodynamic changes. Guo et al. explored the use of a supervised RL LSTM model in 13,762 ICU-admitted patients with coronary heart disease [61]. Their findings indicated that, while RL alone did not significantly reduce in-hospital mortality, it closely mimicked clinician behavior, suggesting its potential as a supplementary tool to enhance clinical decision making. This highlights an important consideration in RL applications: while technology may not yet surpass human expertise in all areas, it can serve as a valuable aid, offering consistency and data-driven insights to support clinician judgment.

Within the field of oncology, diagnosis of certain cancers requires invasive procedures that are often costly and require multiple steps afterwards. Given the complexity of steps within management, developing a strong reinforcement learning model able to make multistage dynamic decisions is invaluable. Tang et al. developed such a model that uses tree-based reinforcement learning to identify optimal test-and-treat options for prostate cancer [62]. Another group attempted to optimize a similar multistep treatment for head and neck cancer using deep Q-learning [63]. The results showed that, by using this approach, survival rates increased by 3.73%. The model’s treatment decisions matched the clinician’s outcome, achieving a mean accuracy of 87.5%. While previous studies explored multistep treatment modalities, Ebrahimi et al. concentrated on addressing the challenges posed by a single fluctuating parameter—the need to continuously adjust the radiation dose during adaptive radiation therapy (ART). ART is a technique that adaptively adjusts the dose of radiation in response to changes during treatment [64]. Ultimately, their goal was to develop an RL framework to optimize these adjustment points to lower drug toxicity and maximize tumor control. The results showed that the new ART treatment plan outperformed the reference plan.

In the field of endocrinology, specifically diabetes, there remains the challenge of optimizing insulin and other medications that can affect patients in real time. Since these treatments are personalized, information might not be readily available for models to use for each patient. Saghafian et al. proposed a model that extends DTR to account for ambiguity from unobserved confounders, introducing learning methods that personalize treatment decisions and demonstrate strong empirical performance [65]. Many reinforcement learning models are trained on retrospective medical records but often perform poorly in offline settings. Despite these challenges, Nambiar et al. developed an offline RL approach that demonstrated the effective optimization of real-world diabetes management while improving consistency with clinical practice and safety guidelines [66]. Additionally, Luckett et al. developed another model that uses mobile technologies to estimate an optimal dynamic treatment regime for glucose levels in patients with type 1 diabetes [67]. This technology leverages mobile tools to allow for outpatient data collection, supporting better control of glucose levels. The results showed that the proposed method can reduce the number of hyper- and hypoglycemic episodes.

The application of reinforcement learning in various medical specialties, including gastroenterology (GI), nephrology, and immunology, is gradually increasing. Within GI, Hu et al. developed REMEDI, a dynamic treatment regime designed to model bile acid dynamics and optimize therapeutic strategies for patients with primary sclerosing cholangitis [68]. Similarly, in nephrology, Abebe et al. introduced a reinforcement learning algorithm that is capable of identifying optimal, multistage, and multi-treatment regimens for individuals with diabetic kidney disease [69]. Within immunology, Liu et al. proposed a deep reinforcement learning (DRL) framework aimed at managing and preventing acute and chronic graft-versus-host disease following transplantation [22]. Although RL-based research in these specialties remains limited, these fields have substantial potential for further advancements through RL-driven methodologies.

Lastly, in the general category of medicine, RL learning has provided dynamic treatment through optimizing the electronic health records (EHR). Sun et al. developed TR-GAN, an offline reinforcement learning model that incorporates real and counterfactual patient trajectories—sequences of states, treatments, and outcomes over time—to optimize treatment recommendations [70]. By leveraging electronic health record data, the model inferred patient states at each time point and demonstrated improved treatment optimization compared to existing offline RL methods. Zhou et al. conducted a study leveraging free-text clinical information to optimize dynamic treatment regimes, demonstrating that incorporating unstructured data improves counterfactual outcome estimation compared to using structured EHR data alone [71]. Their approach resulted in more accurate treatment recommendations, highlighting the value of integrating free-text data into clinical decision-making models. Another study performed by Wang et al. developed supervised reinforcement learning with a recurrent neural network that uses an off-policy actor–critic framework to tackle the intricacies between medication, diseases, and personalized characteristics [72]. In a broader sense of RL and medicine, many patients often present with multiple chronic disease states that cannot be addressed in a singular aspect. Cho et al. developed a reinforcement-learning-based method to optimize dynamic treatment regimes that personalize multistage medical decisions by considering patient-specific factors and survival probabilities [73]. Their approach, utilizing generalized random survival forests, enables adaptive treatment strategies that maximize long-term patient outcomes while addressing challenges such as censoring and variable treatment timing.

### 2.3. Precision Medicine

PM offers an innovative approach to enhance healthcare by enabling tailored interventions that align with a patient’s unique health profile. This methodology utilizes patient-specific data and clinical insights to craft adaptive treatment strategies that optimize outcomes for chronic and complex conditions. Using RL algorithms, PM can achieve high levels of customization in therapeutic decision making, addressing the changing needs of patients with accuracy and efficacy, which enhances both patient outcomes and clinical efficiency. The applications of PM range widely across medical specialties and focus on tailoring treatment plans to individual patients based on their unique characteristics, including genetic makeup, environmental factors, and lifestyle.

Diabetes is an ideal target for PM due to its heterogeneity in lifestyle factors, disease progression, and treatment response. Jafar et al. employed a multi-agent RL algorithm with single-agent RL to optimize insulin bolus recommendations for patients with type 1 diabetes; this fine-tunes glycemic control after high-fat meals and exercise by adjusting insulin doses based on patient responses [74]. This approach demonstrated significant reductions in hypoglycemia and postprandial hyperglycemia by dynamically adjusting insulin strategies to more complex metabolic scenarios. Similarly, Zhu et al. utilized a deep RL algorithm to develop delivery strategies for insulin delivery and dual-hormone (insulin and glucagon) delivery. Both single- and dual-hormone delivery strategies achieved improved glucose control when compared against a standard basal–bolus therapy with low-glucose insulin suspension, as measured by percentage time in target range for both adults and adolescents [75]. In an earlier study, Zhu et al. developed an RL framework for personalized insulin dosing, demonstrating its effectiveness in improving glycemic control compared to standard basal–bolus therapy [76]. Their results showed that the RL-based approach increased the percentage of time within the optimal glucose range while reducing both hyperglycemic and hypoglycemic events, setting the stage for the more advanced dual-hormone strategies explored in their later study. Shifrin et al. implemented Markov Decision Processes (MDPs) with an individualized health reward function that grades blood glucose levels based on patient-specific environmental changes to optimize insulin management [77]. This personalized approach improved blood glucose regulation and demonstrated the potential of reinforcement learning in diabetes care. In addition, Oh et al. used a deep Q-network framework to optimize treatment strategies for patients managing both hypertension and type 2 diabetes. The system recommended treatment regimes ranging from mono to triple therapy, tailored to individual patient profiles. This showed better outcomes in controlling blood pressure and blood glucose levels, while minimizing adverse effects and treatment inefficiencies [78]. In addition, Oh et al. applied Q-learning to personalize antihypertensive regimens in patients with type 2 diabetes and hypertension using South Korean EHR data. The model recommended mono, dual, or triple therapy based on individualized clinical states and achieved higher concordance with physician prescriptions and improved blood pressure control compared to a Markov Decision Process model [17]. Yang et al. developed PrescDRL, a deep RL model designed to optimize herbal prescription planning for chronic disease treatment. It achieved a 117% improvement in single-step reward and a 40.5% increase in prescription precision compared to radiation methods when evaluated on a sequential diabetes treatment dataset [79].

In addition to its application with chronic disease management, RL also has applications within oncology, targeting tailored treatment regimens based on patient-specific tumor characteristics and clinical disease progression. Lu et al. developed a deep RL framework for intermittent androgen deprivation therapy in prostate cancer, using a competition-based model to balance responsive and resistant cells. This method prolonged time-to-progression and reduced drug dosages compared to standard protocols [80]. Eastman et al. trained a deep double Q-learning agent using average patient parameters and relative bone marrow density measurements, which allowed the agent to optimize chemotherapy dosing schedules while minimizing drug toxicity for each patient undergoing chemo [81]. In addition, lung cancer is a prime target for PM due to its variability in tumor characteristics, progression, and patient-specific risk factors, like smoking history and genetic predisposition. Wang et al. uses RL-based policies to individualize lung cancer screening schedules, integrating patient-specific attributes such as nodule size, appearance, and smoking history. These models reduced misdiagnosis, missed diagnoses, and delayed diagnoses compared to guideline-based protocols, demonstrating RL’s capacity for tailoring follow-up intervals to individual risk profiles and enhancing diagnostic accuracy [82]. Niraula et al. utilized reinforcement learning to optimize daily radiation doses for non-small-cell lung cancer and hepatocellular carcinoma patients using the Adaptive Radiotherapy Clinical Decision Support (ARCliDS) system. This model aligned with clinical decisions from physicians in their clinical trial dataset, successfully enhancing favorable decisions in up to 50% of cases while reducing unfavorable decisions in up to 74% of cases, which highlights the ability of RL to refine real-time therapeutic adjustments to improve patient outcomes [83]. Krakow et al. furthers these findings in a more specific context: using Q-learning to optimize immunosuppressive therapy sequences for managing graft-versus-host disease (GVHD) in patients undergoing allogeneic hematopoietic cell transplantation. Their model identified specific treatments associated with improved personalized survival outcomes, which further underlies its potential to refine complex therapeutic strategies [84].

Within critical care, PM addresses highly variable conditions and, by using patient-specific data, enables tailored interventions such as fluid management, ventilatory support, and drug dosing. Shirali et al. uses a multi-objective deep Q-learning model to optimize critical care interventions by leveraging frequently measured biomarker signals with sparse reward structures. Using ICU data, the model enhanced the relatability of critical care policies while maintaining focus on primary outcomes like mortality [85]. Likewise, Ma et al. proposed the Deep Attention Q-Network (DAQN) that integrated historical patient data and improved treatment recommendations for sepsis and acute hypotension. The model demonstrated superior outcomes in managing SOFA scores and lactate levels outperforming alternative methods [86]. Within critical care applications, dialysis treatment has emerged as a target due to its highly variable nature from patient to patient. Grolleau et al. validated an RL-based strategy for initiating renal replacement therapy in ICU patients with severe acute kidney injury, utilizing a policy model that dynamically adjusted based on patient status and increased hospital-free days [87]. Yang et al. applied partially observable MDPs to dynamically adjust dry weight in hemodialysis patients, incorporating real-time patient data to reduce symptoms and improve five-year mortality rates. Electrolyte management in critical care has also benefited from RL innovations [88]. Prasad et al. used fitted Q-iteration to develop a clinical decision support tool, which reduced unnecessary electrolyte repletion by learning optimal repletion policies from historical data, thereby minimizing associated risk [89]. Meanwhile, Weisenthal et al. uses the concept of relative sparsity within RL to optimize vasopressin administration for ICU patients with hypotension, improving mean arterial pressure outcomes through a data-driven policy tailored to patient responses [90]. Feng et al. presented an NIVAI model, an offline RL approach that dynamically recommends optimal noninvasive ventilation (NIV) switching policies for individual patients. This model outperformed physician decisions and reduced mortality rates in high-risk patients by recommending intubation earlier and more often on average than physicians by using partial oxygen pressure, oxygen flow, and Glasgow Coma Scale scores [91]. Wu et al. further advanced PM in critical care by developing the Weighted Dueling Double Deep Q-Network with embedded human expertise, an RL model designed to enhance sepsis treatment decisions. By incorporating clinician knowledge and adaptive Q-value weighting, the model achieved a 97.81% survival rate when tested on the MIMIC-III dataset, outperforming other deep RL approaches [92]. This highlights RL’s potential to optimize management of sepsis in real time, guaranteeing treatment policies are aligned with clinical knowledge and data-driven. Gao et al. developed “Dr. Agent”, a clinical predictive model that mimics the practice of seeking second opinions using two RL agents: the first focuses on the most recent visit of the patient to assess their current status, while the other analyzes the entire patient history comprehensively. Dr. Agent outperformed baseline models when tested on the MIMIC-III database across four tasks, including in-hospital mortality prediction, acute care phenotype classification, physiologic decompensation prediction, and length-of-stay forecasting, showing up to a 15% higher area-under-the-precision-recall curve [93].

The field of cardiology has also embraced RL and is a target for PM with a large range of applications, including refining anticoagulation and rhythm management therapies. Zeng et al. used MDPs to optimize postoperative warfarin anticoagulation, learning from patient-specific data to maintain therapeutic INR ranges and outperform clinician practices [94]. Barrett et al. employed tabular Q-learning to enhance rhythm control in atrial fibrillation for eight specific patient subtypes based on other heart comorbidities, reducing mortality and achieving superior clinical outcomes, including lower mortality and longer time-to-event intervals. Innovative RL models have also targeted personalized warfarin dosing in atrial fibrillation [95]. Petch et al. employed deep Q-learning to optimize dosing algorithms, achieving better time-in-range for INR targets and reducing adverse outcomes such as stroke and hemorrhage by tailoring recommendations to individual patient profiles [96]. Similarly, Zuo et al. utilized RL to enhance anticoagulant treatment strategies, with policies that reduced ischemic stroke and systemic embolism rates by aligning treatments to patient-specific risks [97].

Behavioral health and psychiatry are inherently patient- and situation-specific, making interventions in the field ideal for PM through RL. Piette et al. introduced the “PowerED” program, which used an RL-based model to optimize counselor time allocation by dynamically prioritizing patient needs, resulting in improved opioid misuse scores and more efficient resource utilization [98]. Kahkoska et al. applied RL trees to analyze data from the FLEX trial, identifying subgroup-specific optimal treatment rules for adolescents with type 1 diabetes and improving quality of life metrics through individualized behavioral counseling [99].

### 2.4. Findings of the Review

Individual study results were summarized narratively. No structured summary tables with effect sizes were produced, due to variability in study outcomes and lack of uniform metrics. The findings of the surveyed papers were reported with respect to their type of RL category, RL algorithm, and type of specialty. Figure 5 depicts the number of PM and DTR papers in the study. Since 2015, the number of RL publications per year has been steadily rising, with the largest increase seen in 2020. This indicates the rapid growth and utilization of RL within medicine. Figure 6 shows the type and number of RL algorithms used in each study. Hybrid and value-based RL methods were some of the most-utilized methods, which reflects their ability to optimize sequential decision making while balancing efficiency, adaptability, and interpretability. This is a result of methods’ capability to learn from sparse rewards, optimize long-term patient outcomes, and provide actionable insights for real-world medical decision making. The distribution of RL across different specialties is shown in Figure 7, reflecting the RL algorithm’s diverse applications. A majority of the studies were implemented in critical care and endocrinology, specifically diabetes. Both of these fields benefit from structured and continuous data, which allow RL models, especially PM and DTR, to learn and adapt effectively. Overall, each figure (Figure 5, Figure 6 and Figure 7) demonstrates a sharp increase in publications regarding RL in medicine around 2020–2021. This may have been driven by advancements in computational power, machine and deep learning integration, and increased adoption within healthcare. The COVID-19 pandemic may have also accelerated AI-driven decision making, particularly in critical care where RL models were being used. Moreover, the adoption of personalized and biometric wearable (such as continuous glucose monitors) have provided continuous and real-world data. Across, studies, performance was evaluated using both clinical outcomes (e.g., mortality reduction, glycemic control) and RL-specific metrics (e.g., cumulative reward, policy concordance). A minority of studies also reported statistical performance indicators such as AUROC or predication accuracy. This is represented in Table S3 in the Supplementary Materials. Additionally, we summarized the loss functions used in the included studies, as shown in Table S3. While several studies did not explicitly report their loss function, most used objectives consistent with their RL framework, such as mean squared error (MSE) for Q-function estimation and temporal difference (TD) error in value updates. These loss functions were typically designed to optimize cumulative reward signals relevant to clinical outcomes. Our findings highlight the accelerating adoption of RL in medicine and the growing need for AI-driven decision making within personalized medical care.

### 2.5. Limitations of the Review

This review was conducted using a narrative synthesis approach; thus, no quantitative effect measures were pooled due to heterogeneity in study designs, interventions, and reported outcomes. While this enabled a broader, qualitative analysis, it limits our ability to support statistically supported conclusions. In addition, the scope of the survey was limited to studies indexed in PubMed, MEDLINE, and Web of Science in order to focus on studies that are most relevant to clinical practice. Future reviews could broaden the scope by including additional databases (IEEE Xplore or arXiv) to better capture technical developments and preclinical studies in RL. Non-English studies and unpublished conference proceedings were not included, which may have augmented the review. Formal assessments of study-level risk of bias and publication bias were also not performed. These limitations may affect the comprehensiveness and generalizability of the findings. Future studies should consider incorporating quantitative syntheses to strengthen evidence-based conclusions. Lastly, the review did not include detailed mathematical formulations of RL algorithms, as the review was aimed at clinicians rather than a technical audience.

## 3. Future Directions and Practical Implications

### 3.1. Developing Research Taxonomy for Clinicians

RL has a wide variety of applications within healthcare, including PM and DTR. More research and further clinical implementations in the coming years should aid physicians and the medical field in making clinically relevant decisions. To facilitate this aim, the authors have developed the taxonomy (Table 2) based on their literature review. This taxonomy is separated into challenges and benefits as well as the scope of current applications and the directions of research. In order to develop, implement, and further facilitate appropriate RL use in medicine, let us examine each component more closely.

#### 3.1.1. Challenges

Challenges exist in both the application of algorithms and in the ability to apply algorithms. In some of these challenging areas, especially when dealing with a lack of data, employing advances made in fields such as simulation, could be used to augment known data [100,101]. Challenges related to model interpretability are further compounded by physicians’ limited familiarity with algorithmic methods, making clinical adoption more difficult; the use of explainable artificial intelligence techniques offers a potential solution to enhance understanding and trust [102]. There are also a few ethical implications with the integration of RL within medicine. RL models can perpetuate existing biases if trained on imbalanced or unrepresentative data, potentially resulting in inequitable clinical recommendations. Furthermore, the limited explainability of RL algorithms may also lead to opaque recommendations that lack clinical transparency. Ongoing validation and bias mitigation strategies will be essential to combat this. Lastly, over-reliance on automated decisions can introduce risk if the models are poorly generalized or if the reward function is inadequately defined. As such, these models should augment clinical judgment, not replace it. Therefore, a variety of research questions remain in this area, including the following:

- How can RL methods gain trust for clinician use?
- How can the RL and clinical community better communicate to develop problems of interest and a common lexicon?
- How can clinicians be better educated in algorithms?
- How can research on data augmentation and advanced experimental designs facilitate the use of RL on rare diseases?
- How is RL being applied within clinical decision making and healthcare settings to optimize both quality care and patient safety?

#### 3.1.2. Benefits

Many of the potential benefits are directly related to challenges, e.g., benefits from data-driven outcomes are hampered by a lack of data. Moreover, physicians often rely on clinical skills based on the experience of seeing other patients with a particular condition rather than a specific algorithm. Moving to data-driven medicine provides efficient and dynamic use of data and benefits from using data from a single patient or from multiple patients. A variety of research questions remain in this area, such as the following:

- How can RL benefits be best illustrated and presented to clinicians?
- What metrics and assessment are best suited to enable RL benefits to be provided with minimal harm?
- How can a move toward data-driven patient outcome be best facilitated and welcomed?

#### 3.1.3. Current Applications

The authors largely reviewed the breadth and depth of current applications of RL for medicine in Section 3. However, due to the highly interdisciplinary nature of RL in medicine, the entire scope of current applications might be unknown whereby medical challenge problems are solved in a variety of domains without direct medical collaboration. Thus, research questions in this area include the following:

- What applications are not captured in PubMed due to their interdisciplinary nature and publication in other domains?
- How can challenges posed by medicine-related RL reach medical audiences effectively?
- Can better constructed interdisciplinary teams ensure that medical applications of RL are adequately solved for potential clinical adoption?

#### 3.1.4. Directions

Finally, understanding the direction of RL algorithmic research and having means to rapidly assess algorithms on relevant problems as demonstrated in is key to expanding the use of RL [23,24,29]. Interest in the early exposure to the field of AI and RL in medical school also facilitates its understanding and use [103,104,105]. A variety of research questions remain in this area:

- What are the current research directions in RL that are most applicable to clinical problems?
- What clinical problems should be extended for RL solutions as challenge problems?
- What is needed to best assess RL solutions to clinical problems?

### 3.2. Practical Implications for Clinicians

The rise of digital medicine, bioinformatics, big data, and AI (and its subset, ML) forms a catalyst for the transformation of healthcare. These modalities can improve, modify, and accelerate new discoveries and health outcomes [106,107]. Real-time implementation of RL in clinical settings requires integration with live data sources such as EHR as well as the ability to generate rapid and reliable decisions. Achieving this will depend on developing safe, interpretable, and computationally efficient models that can operate within clinical workflows. Several studies included in this review explored such implementation strategies, including offline RL training and EHR-integrated decision support tools [40,66,71]. While there is strong evidence that AI and related technologies will be useful, the greatest challenge to AI in the healthcare domain is ensuring its adoption within clinical practice. For widespread integration to occur, there must be approval from regulators, adoption from administrators, incorporation within the electronic health records, standardization within protocols and medical applications, and funding. Furthermore, it will ultimately require not only buy-in from physicians but also successful education of how to utilize the AI modality and subsequent integration within their clinical practice [104,108]. As prior technological advancements have shown, these challenges will eventually be overcome, but likely at a slower rate than the evolution of the technology themselves. Importantly, it is clear that AI will augment healthcare and propel its evolution, as opposed to replacing human clinicians. In the future, this will allow humans more time for data analysis versus data entry, and ultimately transform our scope of practice to prioritize human skills, including empathy, motivation, strategic overview, and overall perspective [109]. In order for clinicians to understand and apply RL in medicine, there is a need to comprehend its basics, potential applications, benefits, and challenges. As previously discussed, we have reviewed the basics of ML (including RL), highlighted its applications within PM and DTR, and developed a taxonomy to provide a structure for clinicians to start to understand and implement RL. Importantly, it is critical to understand RL’s weaknesses and strengths to effectively integrate one’s practice with it. Therefore, as we have detailed in Table 3, we have compiled a concise list of the practice know-hows clinicians should be cognizant of. It is our hope that clinicians can use these frameworks to begin their own transformation of understanding and utilizing AI. Lastly, cognizance of future avenues is advantageous for clinicians to anticipate how RL may evolve in tandem with other technologies. For example, hybrid approaches that combine RL with emerging deep learning architectures, such as vision transformers (ViT) and large multimodal models, have demonstrated superior performance in pattern recognition and representation learning compared to traditional models [110]. These architectures could enhance RL’s ability to interpret complex clinical data and support more-informed decisions.

## 4. Conclusions

RL faces many challenges in its progress toward implementation. However, the potential advantages of RL as an enabler of PM are incredible. To facilitate the appropriate and wider use of RL in clinical settings, the authors first reviewed RL, its trends and current applications in medicine. From this, the paper develops a taxonomy of RL use in medicine and poses further research questions that can improve the use of RL for clinical applications in both outpatient and inpatient practices. The most notable challenge facing implementation of RL is the lack of clear standards in what the algorithm should value as its reward. To that effect, it is up to clinicians and patients to determine and build appropriate RL algorithms to reflect their own goals. Likewise, these RL algorithms need to be transparent to the point clinicians are able to decipher the inputs that went into building them and the way the particular RL defines reward. The applications that lead to the implementation of RL need to be easy to use by physicians at any level of training from a novice physician that lacks an understanding of RL to an already overburdened physician. Inevitably, technological problems such as limited computational power and lack of data availability for many pathologies will be overcome in time. Lastly, for clinicians, the implementation of RL within the clinical setting is rapidly expanding. RL’s ability to learn from real-time patient data and optimize dynamic treatment plans make it a powerful and auspicious paradigm for strengthening personalized medicine. By ceaselessly adapting to a patient’s treatment response, RL can propel more effective and precise healthcare interventions that optimize outcomes while minimizing risks and side effects. This approach of utilizing RL to enhance PM and DTR represents a needed shift from static treatment protocols to dynamic and personalized strategies that evolve with the patient’s condition. The application of RL and other ML strategies within healthcare may ultimately be the catalyst in the next evolution of medicine—a paradigm that effectively utilizes big data to augment patient care.

## Figures and Tables

**Table 1:** Categories of the applications of RL in medicine.

Categories of Reinforcement Learning in Medicine	
(1) Precision Medicine (2) Dynamic Treatment Regime (3) Clinical Support Systems (4) Medical Imaging (5) Diagnostic Systems (6) Dialog Systems	(7) Personalized Rehabilitation (8) Control Systems (9) Health Management Systems (10) Drug Discovery and Development (11) Robotic-Assisted Surgery (12) Wearable Devices and Remote Patient Monitoring

**Table 2:** General taxonomy of reinforcement learning for clinical applications.

Taxonomy of Reinforcement Learning for Clinical Applications	
Challenges	Benefits
Compute power Lack of data Defining rewards Understandability	Dynamic Efficient Data-driven outcomes
Current Applications	Directions
Precision medicine Dynamic treatments Clinical support Diagnostic systems Medical imaging	Intervention delivery optimization Individual treatment plans Manage complex disease over time

**Table 3:** RL aspects and practical know-hows for clinicians.

Aspect	Practical Know-How
RL Basics	RL learns through interactions by using rewards and penalties to optimize decisions.
Data Familiarity	Know the importance of high-quality data and how RL uses patient-specific variables for PM.
Integrations	RL offers real-time recommendations. Use clinical judgment to interpret and validate suggestions.
Patient Safety and Ethics	Prioritize patient safety by ensuring treatments guided by RL are safe. Ensure transparency in the RL application to encourage patient trust and shared decision making.
Limitations	Be aware of data limitations and biases in RL models, including the risk of overfitting. Critically evaluate model performance across diverse and heterogeneous patient populations.
Collaborations	Work with administrators, regulators, and data scientists to align the RL models with clinical priorities, ensuring patient safety, health outcomes, ethical considerations, and regulatory compliance.
Training and Education	Engage in continuous education to learn AI/ML concepts and develop critical evaluation skills to assess RL models in clinical practice.

## Data Availability

No new data were created or analyzed in this study. Data sharing is not applicable to this article.

## Supplementary Materials

The following supporting information can be downloaded at: https://www.mdpi.com/article/10.3390/healthcare13141752/s1, Table S1. Sample of Excluded Studies and Rationale. Table S2. GRADE Framework Summary. Table S3. Summary Table of Dataset.

## Abbreviations

The following abbreviations are used in this manuscript:

AI	artificial intelligence
ML	machine learning
RL	reinforcement learning
PM	precision medicine
DTR	dynamic treatment regimen
PRISMA	Preferred Reporting Items for Systematic Reviews and Meta-Analyses
GRADE	Grading of Recommendations, Assessment, Development, and Evaluation
PD	Parkinson’s disease
LSTM	long short-term memory
SUD	substance use disorder
BOWL	backward outcome-weighted learning
SOWL	simultaneous outcome-weighted learning
ICU	intensive care unit
MDP	Markov Decision Process
GORL	goal-oriented reinforcement learning
ART	adaptive radiation therapy
GI	gastroenterology
DRL	deep reinforcement learning
EHRs	electronic health records
NIV	noninvasive ventilation
