# Supplementary material for: Reinforcement Learning and Its Clinical Applications Within Healthcare: A Systematic Review of Precision Medicine and Dynamic Treatment Regimes
Source: Healthcare (Basel). 2025 Jul 19;13(14):1752. doi: 10.3390/healthcare13141752 (PMC12295150; doi:10.3390/healthcare13141752)
Supplement: Supplementary file 1 [file healthcare-13-01752-s001.zip › Supplementary Tables S1-S3.pdf]

Table S1. Sample of Excluded Studies and Rationale.

Exclusion Category	Study	Reason
Priority Focus on other Machine learning algorithms	Petersen, B. K., Yang, J., Grathwohl, W. S., Cockrell, C., Santiago, C., An, G., & Faissol, D. M. (2019). Deep Reinforcement Learning and Simulation as a Path Toward Precision Medicine. *Journal of computational biology : a journal of computational molecular cell biology*, *26*(6), 597–604. https://doi.org/10.1089/cmb.2018.0168	Focuses on simulation-based deep RL without clinical validation
	Marriott H, Kabiljo R, Hunt GP, et al. *Unsupervised machine learning identifies distinct ALS molecular subtypes in post-mortem motor cortex and blood expression data.* Acta Neuropathol Commun. 2023;11(1):208. https://doi.org/10.1186/s40478-023-01686-8	Applies unsupervised ML, not RL
Insufficient data size for algorithm training and validation	Chen P, Dong W, Lu X, et al. *Deep representation learning for individualized treatment effect estimation using electronic health records.* J Biomed Inform. 2019;100:103303. https://doi.org/10.1016/j.jbi.2019.103303	Uses limited EHR data without large-scale validation
Focus on alternative RL categories	Zhou N, Wang L, Almirall D. *Estimating Tree-Based Dynamic Treatment Regimes Using Observational Data with Restricted Treatment Sequences.* Biometrics. 2023;79(3):2260–2271. https://doi.org/10.1111/biom.13754	Uses tree-based RL, not canonical RL frameworks
Other publication types (reviews, editorials, meta-analyses)	Venkatesan KB, Benjamin LS, Satchi NS. *Reinforcement Learning in Personalized Medicine: A Comprehensive Review of Treatment Optimization Strategies.* Cureus. 2025;17(4):e82756. https://doi.org/10.7759/cureus.82756	Narrative review
	Zhang Z, AME Big-Data Clinical Trial Collaborative Group. *Reinforcement learning in clinical medicine: a method to optimize dynamic treatment regime over time.* Ann Transl Med. 2019;7(14):345. https://doi.org/10.21037/atm.2019.06.75	Editorial

Table S2. GRADE Framework Summary.

Table S2 is submitted has Microsoft Excel document due to its size.

Table S3. Summary Table of Dataset.

Table S3 is submitted has Microsoft Excel document due to its size.
